# Gender-based violence: Statistical data for four Colombian municipalities

**DOI:** 10.1016/j.dib.2022.108320

**Published:** 2022-05-28

**Authors:** Natalia Escobar-Váquiro, Lina Buchely-Ibarra, Sandra Balanta-Cobo

**Affiliations:** a Universidad Icesi, Measurement coordinator of the Observatorio para la Equidad de las Mujeres, Colombia; b Universidad Icesi, Director of the Observatorio para la Equidad de las Mujeres, Colombia; c Universidad Icesi, Researcher of the Observatorio para la Equidad de las Mujeres, Colombia

**Keywords:** Violence against women, Valle del Cauca, Microdata, Cali, Buenaventura, Yumbo, Jamundí

## Abstract

In this article, we describe the dataset on the conditions for gender-based violence (GBV) for women in four municipalities of Colombia: Cali, Buenaventura, Jamundí, and Yumbo. The database was developed by the Observatory for Women's Equity (OEM), an entity resulting from an alliance between Universidad Icesi and Fundación WWB Colombia. The OEM's purpose is to construct measurements that make it possible to account for GBV suffered by women. The following types of violence were classified: psychological violence, physical violence, sexual violence, workplace violence, and economic violence. In addition to the module on GBV, the survey has other modules with which to establish a socioeconomic characterization of women and households, through which to identify how these conditions can be linked to GBV. The sample size was 1,593 women in the four mentioned municipalities.

## Specifications Table

**Table:** 

Subject	*Gender studies*
Specific subject area	Gender-based violence
Type of data	Text, ordinal, nominal, and interval-ratio variables
How the data were acquired	Through telephone survey
Data format	Raw data
Description of data collection	The sample was applied in four municipalities of Valle de Cauca: 1) Cali, 2) Jamundí, 3) Buenaventura, and 4) Yumbo. We implemented a multi-stage probability sampling method to select units by simple random sampling. The sample was stratified by political division and socioeconomic strata. The political division was defined as follows: (1) Extended center and peri‑center zone (communes 3, 4, 8, 9, 10, 11, and 12); (2) Ladera zone (communes 1, 18, and 20); (3) North-south corridor urban zone (communes 2, 5, 17, 19, and 22); and (4) East urban zone (communes 6, 7, 13, 14, 15, 16, and 21) in the case of Cali; and by comunas for Yumbo, Jamundí, and Buenaventura.
Data source location	Cali, Buenaventura, Yumbo, and Jamundí- Valle del Cauca, Colombia. Cali: 3°27′00″N 76°32′00″O Buenaventura: Latitude 3,5,833,299 and longitude −77, in the northern hemisphere. Yumbo: latitude 3.58234 and longitude −76.4914627, in the northern hemisphere. Jamundí: Longitude: O76°32′12.98″ Latitude: N3°15′38.99″
Data accessibility	Repository name: Mendeley Data. Data identification number: doi: 10.17632/d37kcsp8jg.3 https://data.mendeley.com/datasets/d37kcsp8jg/3

## Value of the Data

• The information collected for this survey incorporates an intersectional and gender perspective. The survey inquires about the individual characteristics of the women including ethnicity, sexual orientation, occupational category, educational level, income level, etc. In addition to investigating the incidence of each of these types of violence, the survey explores the perpetrator of the violence, whether it has been suffered several times or only once, and how it has occurred over time, taking into consideration the last week, the last month, the last year, whether it was more than a year ago or over 10 years ago.
• Violence against women or gender-based violence is a violation of human rights and a major public health problem because of the implications it has on the physical and mental well-being of women and its social costs [1]. This makes it a problem that urgently needs to be addressed through better public policies. In turn, these better policies require, among other things, better sources of information, and despite the seriousness of the problem, there is a gap in the collection of information associated with gender-based violence [2], [3].
• For this reason, the data collected in this survey are of interest to GBV scholars considering that they can be explored taking into consideration intersectional factors that may exacerbate the incidence of GBV such as ethnicity, sexual orientation, education level, occupational category, etc. Demographic and Health Surveys (DHS) provide important information on these forms of violence and are available for the vast majority of countries but have limitations because they only consider women up to 49 years of age. This limitation has already been pointed out by entities such as the WHO [4], which has highlighted the need to disaggregate by age and include women over 50 years of age in the survey.
• Although this survey does not allow national-level comparisons because most of the information on GBV comes from administrative records such as complaints to the prosecutor's office, the forensic medicine institute, or data collected from telephone lines, it does offer the possibility of comparing four municipalities in one of Colombia's regions, which participates significantly in the number of cases of different types of violence against women in the national total. Finally, it also allows quantitative approximations to identify the variables or factors that can help explain GBV.

## 1 Data Description

The data presented in this survey was collected from August 26 to October 26, 2020. The questionnaire was designed by the observatory's measurement team and reviewed by internal and external peers from Universidad Icesi who have expertise in gender issues and in statistical operations. The women answered questions associated with socioeconomic characterization, gender-based violence, and economic and financial autonomy.

The study's unit of analysis is made up of women of legal age (18 years, in Colombia). The sample was structured in four municipalities of Valle del Cauca: Santiago de Cali, Buenaventura, Yumbo, and Jamundí. A multistage stratified probability sampling was implemented with a selection of units by simple random sampling. Socioeconomic strata were used as the main stratification variable for Buenaventura, Yumbo, and Jamundí, whereas for Cali, we used zones, defined as follows: Extended center and peri-center zone (communes 3, 4, 8, 9, 10, 11, and 12); (2) Ladera zone (communes 1, 18, and 20); (3) North-south corridor urban zone (communes 2, 5, 17, 19, and 22); and (4) East urban zone (communes 6, 7, 13, 14, 15, 16, and 21). In the first stage, the households were selected via the telephone sampling frame, and in the second stage, a person within the household was selected to answer the survey (Only one woman per household is selected). Table 1 presents the sample by socioeconomic stratum^1^ or socioeconomic level in an absolute fashion and the expanded sample for the four municipalities.

**Table 1:** Absolute and expanded frequency of units by socioeconomic level (socioeconomic stratum) by municipality.

Municipality	Socioeconomic level 1	Socioeconomic level 2	Socioeconomic level 3	Socioeconomic level 4	Socioeconomic level 5	Socioeconomic level 6	Total
	Sample						

Cali	96	130	154	49	42	19	490
Buenaventura	189	68	58	6			321
Jamundí	32	215	90	39	14	2	392
Yumbo	91	247	49	2	1		390
Total	408	660	351	96	57	21	1593

	Expanded sample^a^						

Cali	166,780	234,278	284,793	97,532	72,963	38,991	895,337
Buenaventura	44,223	17,461	15,853	1001			78,538
Jamundí	4177	27,597	11,618	5045	1852	276	50,565
Yumbo	8464	22,666	4486	164	92		35,872
Total	223,643	302,003	316,750	103,742	74,907	39,267	1,060,312

a Since this sample corresponds to a probabilistic sampling that seeks to make an inference from the total population of women in the four municipalities, a final expansion factor is calculated using the unbiased Horvitz-Thompson estimator: ${\hat{t}}_{y,\pi }=\sum_{s}\frac{y_{k}}{\pi_{k}}$Where $\pi_{k}$ is the probability of inclusion for the k-th final sampling unit. “S” indicates that the sum is performed on the random sample. So when we refer to an expanded sample we refer to the results of the inference for the entire population and not just for the sample.

The municipalities have chosen by OEM observatory target the subnational level. The survey has been focused on Valle del Cauca since 2018, specifically in the municipalities of Cali (the capital of Valle del Cauca), Yumbo, Jamundí, and Buenaventura. These municipalities were selected strategically. Cali, Jamundí, and Yumbo are close to each other, and they have shared economies, businesses, and mobility. They make up the metropolitan area of ​​Santiago de Cali, so having data from the three municipalities will help us understand the overall dynamics of the largest and most important city in Valle del Cauca.

Buenaventura, on the other hand, is the point of contrast. Geographically located on the Pacific coast, it is the second most important port in Colombia, with high poverty, inequality, and violence indicators. It was also the worst-hit city in Valle del Cauca by the armed conflict and a national reference point for public policy advocacy in the Pacific. This city was selected by the survey's funders as the second city prioritized for work (the first being the capital, Cali).

The questionnaire contains 256 questions distributed as follows: sociodemographic characteristics, household characteristics, gender-based violence, and economic and financial autonomy. The duration of data collection averaged 28 min. Raw data and supplementary material are attached. Table 2 presents the general characteristics of the women surveyed. These results are for the unexpanded sample as well as for the rest of the tables in which results are presented.

**Table 2:** General characteristics of the sample.

Variable	%
**Municipality**	
Cali	30.76
Buenaventura	20.15
Jamundí	24.61
Yumbo	24.48
**Total**	**100**
**Socioeconomic stratum of household**	
One	25.61
Two	41.43
Three	22.03
Four	6.03
Five	3.58
Six	1.32
**Total**	**100**
**Age group**	
18 - 24	14.38
25 - 34	20.28
35 - 44	20.40
45 - 54	19.96
55 - 64	15.32
Over 65	9.67
**Total**	**100**
**Ethno-cultural self-identification**	
Afro-Colombian, Afro-descendant (black, mulatto, root, palenquera)	37.92
Indigineous	2.76
Mestiza	29.76
White	17.51
Rom o gypsy	0.06
Other	2.51
None	9.48
**Total**	**100**
**Marital status**	
Single	38.04
Free union	28.25
Married	21.34
Separated / Divorced	7.03
Widow	5.08
Other	0.25
**Total**	**100**
**Education level**	
None	0.88
Elementary School incompleted	7.28
Elementary School completed	8.66
High School incompleted	12.81
High School completed	29.13
Technological or Technical studies inco	3.58
Technological or Technical studies comp	21.28
Bachelor's Degree incompleted	3.70
Bachelor's Degree completed	7.22
Postgraduate incompleted	0.38
Postgraduate completed	5.08
**Total**	**100**
**Occupational categories**	
Unpaid housework	43.63
Working	30.82
Looking for a job	15.07
Studying	5.84
Permanently disabled for work	2.45
Other activity	2.20
**Total**	**100**
**Household type**	
Extended	43.75
Married couples with children	23.67
Single parent	12.87
Single person	6.59
Married couples without children	6.47
Multiple family	5.90
Non-family	0.75
**Total**	**100**

Finally, it is important to note that the response rate for this survey was 72% (a total of 2213 calls were made and a total of 1593 surveys were completed).

## 2 Experimental Design, Materials and Methods

To design the survey, we first searched for national and international surveys that address gender-based violence, not only from its instrument but also based on feminist methodologies that make it possible to maintain an ethical and safe approach for the respondents. From this search, the following measurement experiences were specifically considered:

• National survey of violence against women 2006, Mexico [5]: this survey is carried out on women users of the Mexican health system. In this sense, the modules on sexual and physical violence by partners or ex-partners were essential for the construction of the survey.
• Health and demographic survey 2015, Colombia [6]: although this is a broad survey on health and sexuality, it has a very good module on gender-based violence that also presents the definitions that were addressed in our study, that is how this module of this survey was used to construct the OEM survey.
• Sexist violence survey 2016, Cataluña [7]: this survey represents a significant advance for the measurement of gender-based violence since it includes the advances that have been made in other regions on this issue, both in operational and epistemological terms. In our case, this survey helped us to structure violence in the workplace and to measure violence by people who are not a partner. This last part focused on the recognition of the actors who exercise violence outside the couple.
• National prevalence survey on gender-based violence and generations 2020, Uruguay [8]: This survey differentiates between what happens in the public and private spheres. In this sense, two modules were particularly useful for the construction of our survey: violence in the work environment that is part of the public sphere and violence in the context of the couple that is part of the private one.
• National survey on violence against women in France 2003, France [9]: This is the first survey on violence against women that is representative of a country. In our case, it was especially useful in two areas, sexual and economic violence. Additionally, this survey shows us a methodological path to measure the prevalence of GBV.

Based on the above, five modules were designed to address the types of violence: psychological, physical, sexual, economic, and patrimonial, and violence at work. The questions were designed according to each type of violence and are adaptations or adjustments to the country context. In the first four modules, the question structure investigates the victimizing events in four dimensions: i) occurrence: whether the event happened or not, ii) victimizer: a person who perpetrated the event, iii) incidence: number of times that the event has occurred, iv) prevalence: when was the last time the victim was subject to the incident. In the last module on violence at work, the questions refer only to the occurrence of violent events in the workplace.

These modules on gender-based violence are accompanied by three other modules. The first two are on the socioeconomic characterization of the respondents and the characterization of the households. The last module of the survey is on economic autonomy. The dimensions addressed made it possible to identify the link between the respondents’ social and economic conditions and GBV.

The survey modules include eight modules that are organized as follows:

• Sociodemographic information: In this section, we explore basic information about women associated with their sociodemographic position.
• Household: This section seeks to collect information on kinship relationships within households.
• Gender-based violence: This section is made up of 4 modules whose conceptualization is the framework of what *Profamilia* [10] has defined in each of these types of violence:
  ○ Psychological violence: “This includes any action or omission intended to degrade or control the actions, behaviors, beliefs, and decisions of other people through intimidation, manipulation, threat, humiliation, isolation, or any conduct that involves damage to psychological health. This type of violence is one of the most common and naturalized in society, so we must learn to recognize and report it.”

For the construction of this section, the following 6 questions were used (Table 3).

**Table 3:** Results of psychological violence.

Question	Yes	NA
Someone has made comments to belittle you or diminish your self-esteem (e.g., You are a stupid, you are good for nothing, you don't do anything right, etc.)?	25.24	0.13
Has anyone made unwanted comments to you about your appearance or body that have made you feel uncomfortable or insecure?	25.05	0.13
Has anyone sent you messages, posted comments with non-consensual sexual innuendos via cell phone, email or social networks, published photographs or private information about you without your consent, on networks or threatened you with doing so, etc.?	8.79	0
Has anyone stalked or followed you when you left school, home or work?	7.91	0.06
Has anyone prevented you from meeting with friends or tried to limit contact with your family?	6.59	0.13
Has anyone threatened you and/or taken you away from your loved ones or pets (include if your loved ones or pets have been hurt)?	6.72	0.13

Asking questions about GBV implies that we must have special considerations to prevent women from being re-victimized. In this sense, all the designed modules that directly address this topic include within the question options: does not want to answer. For this reason, in Table 3, Table 4, Table 5, Table 6, Table 7, Table 8, the NA column is included in the results, which shows the percentage of women who did not want to answer these questions.

**Table 4:** Structure of derived questions.

Question	answer options	Indicator type
Who?	Any of your parents	This makes it possible to recognize the actors who exercised the violence based on their family or social relationship.
	Any of your children	
	Other relative	
	Your current partner	
	Your former partner	
	A non-relative acquaintance	
	A stranger	
	Do not know/no answer	
Has it happened several times or just once?	Many times	This question makes it possible to recognize recidivism in acts of violence
	One time	
	Do not know/no answer	
When was the last time this happened to you?	More than 10 years ago	This question establishes the prevalence of violence over time. In addition to making a recognition of these during the period of mandatory confinement caused by Covid19.
	More than a year ago	
	During the last year	
	During the last month	
	During the last week	
	Do not know/no answer

**Table 5:** Results of economic violence.

Type	Question	Yes	NA
General	Has anyone restricted your access to resources such as money or food?	5.27	0.06
	Has anyone appropriated or taken money or goods (property, land) from you?	7.60	0.31
	Has anyone forbidden you to study or work or start a business?	4.90	0.06
	Has anyone barred you from claiming any allowances or if you have received them have they been taken away?	1.13	0.19
	Has anyone hidden or controlled your documents with which you could have access to resources such as ID cards, bank cards, pass, among others, or has prohibited you from having a bank account?	1.00	0
	Has anyone threatened to throw you out of the house if you does not agree to their requests?	5.59	0.13
	Has anyone demanded that you hand over the money you earned?	1.51	0.06
	Did your partner or ex-partner put all assets in his name (e.g., house, car, cell phones) or has he sold your assets or household assets without your consent?	4.69	0.07
Vicarious (46.6% of women have children who depend economically on them)	Has your partner or ex-partner - having the money - refused to pay your children's school expenses?	21.33	0.26
	Has your current or ex-partner-having the money-refused to buy clothes or household goods for your children?	17.62	0.38
	Has your current or former partner-having the money-refused to buy food for your children?	14.18	0.51
	Has your current or ex-partner - having the money - refuse to pay child support?	18.01	1.02
Partner	Has your current or ex-partner monitor what you do at work (time of arrival and departure, activities you do, relationships with co-workers, etc.)?	6.41	0.59
	Has your current or former partner make important decisions about how to use money in the household without consulting you or taking your opinion into account?	8.00	0.46
	Has your current or former partner destroyed your belongings?	7.67	0.07

**Table 6:** Results of sexual violence.

Question	Yes	NA
Has anyone ever shown you their private parts or touch them in front of you?	16.01	0
Have you been forced to watch sexual or pornographic scenes or acts?	1.13	0
Have you been propositioned or approached for sex in exchange for any benefits?	14.19	0.06
Have you been punished, mistreated, assaulted, or taken any action against you because you refused to have sex?	4.71	0.19
Have you been groped, touched, kissed, or approached, touched, kissed intentionally and without your consent?	20.53	0.06
Have you been forced to have sex against your will, even if you initially consented to the relationship and then changed your mind?	8.16	0.31

**Table 7:** Resultados de physical violence.

Question	Yes	NA
Have you been kicked, hit with a fist, pinched, had your hair pulled, pushed, shoved, pulled, slapped, or thrown an object?	22.03	0.13
Have you been attacked or assaulted with a knife, firearm or chemicals?	7.47	0

**Table 8:** Results of violence at work.

Question	Yes	NA
Have you been paid less than a fellow man who does the same job or has the same position as you?	23.59	1.66
Have you had fewer opportunities for promotion compared to men?	29.38	1.09
Because of you are pregnant, or because you have small children, you have not been hired, your salary has been lowered or you have been fired?	20.04	0.87
Because of your marital status, gender identity or sexual orientation, you were not hired, your salary was lowered or you were fired?	5.50	0.80
Have you ever been asked to take a pregnancy test as a requirement for employment or continuation of employment?	27.57	0.58
Have you been denied maternity leave law or breastfeeding leave?	2.53	0.29
Have you ever been told that because you are a woman you are not suitable for a job?	14.69	0.29
Have you been forced to work longer hours than your male colleagues?	8.61	0.36
In your job have you had to perform unpaid care work activities?	14.47	0.36
Have you ever reported any of these situations of violence?	14.07	0

Additionally, each of the questions in which they resulted in a Yes were asked (Table 4).

○ Economic violence: “This occurs when money is used as a factor to dominate or establish damaging power relationships. This type of violence can manifest itself when money is taken away from a person, who is prevented from spending it for his or her own benefit and that of his or her family, or denied money to control his or her independence.”

In this section, 15 violence recognition questions were used, divided into three parts (Table 5). First, it asks about economic violence in general and that it could involve various actors (8 questions). A second part that asks about vicarious violence, that is, the violence that is exerted towards the couple's children with the intention of harming the couple (4 questions). And a third part that only focuses on violence within the couple (3 questions).

In this case, the questions about actors, prevalence, and incidence are added only to those that respond to general economic violence. In the case of vicarious violence, questions are asked about temporality and incidence, but not about the actors. And in the case of violence in the context of the couple, the question does not ask additional questions.

○ Sexual violence: “This includes all sexual or verbal relationships or acts, not desired or accepted by the other person. Men or women can fall victim to sexual violence when force, physical or psychological coercion, or any other mechanism that nullifies or limits personal will is used against them.”

As with psychological violence, there are 6 questions on sexual violence (see Table 6) and the structure of derived questions is the same.

○ Physical violence: “This includes all attacks to a person's body, whether through blows, throwing objects, confinement, shaking, or squeezing, among other behaviors that may cause physical damage.”^2^

In the case of physical violence, two questions are asked and the structure of the derived questions is the same as in the case of sexual and psychological violence (see Table 7).

• Violence at work: The aim here was to characterize acts that can be configured as violence at work based on the definition of the International Labor Organization (ILO) [11] that defines it as “Any action, incident or behavior that deviates from what is reasonable through which a person is attacked, threatened, humiliated or injured by another in the exercise of his professional activity or as a direct consequence of it.”^3^

Violence at work is measured with 9 general questions and an additional question to measure the level of reporting of this violence (see Table 8).

Economic and financial autonomy: This autonomy is conceptualized from the perspective of ECLAC, which means that financial and economic autonomy will be understood as a broad notion that refers to the capacity and material conditions that women need in order to have effective control over their own lives. This general sense of financial and economic autonomy combines two analytical concepts and three thematic categories. Being an autonomous woman in economic-financial terms means having a certain level of financial independence that makes it possible to make decisions that there are reasons to value. It also involves having some participation or control over the decisions involving the allocation, distribution, and enjoyment of resources. Financial independence means having the means to support oneself economically while maintaining a good quality of life, and to freely decide the destination of your income without requiring the authorization of a third party.

### 2.1 Telephone interviews

The survey was conducted by telephone due to the mandatory confinement imposed by the Covid-19-related restrictions in Colombia at the time of application. Telephone surveys have some limitations compared to face-to-face surveys, including high rates of abandonment and rejection of the survey by this means, which in Colombia is a mechanism used by criminals for theft. To compensate for this situation, the questionnaire was shortened so that data collection did not exceed 30 min and, on the other hand, an ethical and biosafety protocol was designed so that the women would feel in a more secure environment. However, the survey by this means also offers benefits and in the particular case of this survey that inquired about GBV, it affords some women a sense of anonymity to answer questions that may be very sensitive.

For the fieldwork of this statistical operation, the National Consulting Center -CNC^4^- (a company dedicated to carrying out social, business, and market studies that have extensive experience in the development of this type of survey) was contracted. The sampling frame available in the CNC is a telephone directory with about 6.9 million landline numbers and 10 million cell/mobile numbers that are constantly updated.

In the final base of this study, it has 55% cell/mobile registrations and 45% landline numbers. In the case of Colombia, the calls received are not charged, that is, the people who answered the survey did not have any charge. In the case of the cost assumed by the CNC for the year 2020, the cost per minute to a cell phone number was 50 COP (0.01USD) while the minute to landlines was 30 COP (0.008USD). The selection of telephone records is random; It works through a record download algorithm used by the Call Center of the CNC.

### 2.2 Informed consent

Since the survey process was carried out by telephone, informed consent was included at the beginning of the interview. Although the complete questionnaire can be consulted in the Mendeley repository in which the data and other materials of this survey were deposited, here is the section referred to:

“Good morning. My name is ______ of the Centro Nacional de Consultoría, a private company dedicated to market, social and public opinion research, and work for the Observatorio para la Equidad de las Mujeres, of the ICESI university and WWB Foundation Colombia. We are conducting a citywide survey of women over the age of 18 on issues associated with family life, the economy, public participation and gender-based violence”.

This information will be used only for statistical and academic purposes to influence policies in favor of women's equality. This is in accordance with Law 1581 of 2012 on the protection of personal data. All information that you provide us will be kept strictly confidential and will not be disclosed to others. Your participation in this interview is voluntary and if any question arises that you do not want to answer, let me know and I will continue with the following questions.

We hope to count on you, since your participation is very important for this process. ¿Do you agree? With these details, I ask for your authorization to take your data and do the survey, which will take us approximately 20 min.

Authorize: Yes______ No______

Additionally, this survey displays an ethics protocol that is explained below.

### 2.3 Ethics protocol

The literature review and previous experiences conducted as part of the research showed that this type of measurement process where GBV is investigated, can put women at risk if they happen to be responding in spaces which they share with their partner or aggressor. Hence, the importance of training and protocols designed to reduce these negative impacts [12]. In addition to these risks, conducting the survey in times of pandemic implied biosecurity measures, so the survey was conducted by telephone and, at the time of application, the country's confinement measures were based on population groups, which meant that for it was difficult for some women to find time alone so as to be at easr when responding.

Given the above, it was necessary to design an ethics protocol that would protect respondents and interviewers. The interviewers received training that, in addition to including everything related to the questionnaire and the ethics protocol, included conceptual aspects about gender and GBV. They were also trained to practice emotional restraint. Considering that the respondent could be close to her possible aggressor, it was defined that the woman could name a fruit whenever she felt at risk, and with this signal, the interviewer would understand the situation of possible danger to which she would be exposed. Once this happened, the interviewer would stop asking questions and wait for an indication from the respondent that they could continue the interview. If the situation in which the woman surveyed involved greater risks, the woman would say the name of a fruit and hang up. The interviewer would then wait 5 min to call back and if the call was not answered, the interviewer would proceed to call the local authorities in charge. All this implied that the OEM, as the entity in charge of the survey, activated GBV attention routes with the women's or gender equality offices in the municipalities where the surveys were being administered. A virtual channel was also established and constantly reviewed by the OEM staff, to activate the route in a timely manner whenever required.

## Ethics Statements

The ethics committee of the Icesi University has classified the research of the Observatory for Women's Equity as low risk. All procedures are in accordance with the code of ethics of the Icesi University and the respondents gave their consent for the use of the information in accordance with Law 1581 of 2012 on data protection.

## CRediT authorship contribution statement

**Natalia Escobar-Váquiro:** Conceptualization, Methodology, Validation, Formal analysis, Investigation, Resources, Data curation, Writing – original draft, Writing – review & editing, Visualization, Supervision, Project administration. **Lina Buchely-Ibarra:** Conceptualization, Methodology, Validation, Investigation, Resources, Writing – original draft, Writing – review & editing, Visualization, Funding acquisition. **Sandra Balanta-Cobo:** Validation, Formal analysis, Investigation, Resources, Data curation, Writing – original draft, Writing – review & editing, Visualization.

## Declaration of Competing Interest

The authors declare that they have no known competing financial interests or personal relationships that could have appeared to influence the work reported in this paper.

## Data Availability

Gender-based violence: Statistical data for four Colombian municipalities (Original data) (Mendeley).

## Appendix Supplementary materials

Image, application 1

Image, application 2

## Footnotes

1 Socioeconomic stratification is a classification used in Colombia that emerged in the 1980s as a method for the presumption of subsidies in public services. There are 6 strata, where 1 corresponds to the households with the greatest deprivation and 6 the households with the best socioeconomic conditions. The document Conpes 22 of 1994 also helped it as a method of geographical targeting for the protection of social spending and with it, it became a mechanism that allows us to approximate the wealth or poverty of households.

4 https://www.centronacionaldeconsultoria.com/.

## Footnotes

2 Free translation from: https://profamilia.org.co/aprende/violencia-de-genero/tipos-de-violencias/.

3 Free translation from: https://www.oitcinterfor.org/taxonomy/term/3510.
