# Supplementary material for: Gender-based violence: Statistical data for four Colombian municipalities
Source: Data Brief. 2022 May 28;43:108320. doi: 10.1016/j.dib.2022.108320 (PMC9189776; doi:10.1016/j.dib.2022.108320)
Supplement: Supplementary file 1 [file mmc1.pdf]

OEM
OBSERVATORIO PARA LA EQUIDAD DE LAS MUJERES

BIOSECURITY AND ETHICS PROTOCOL

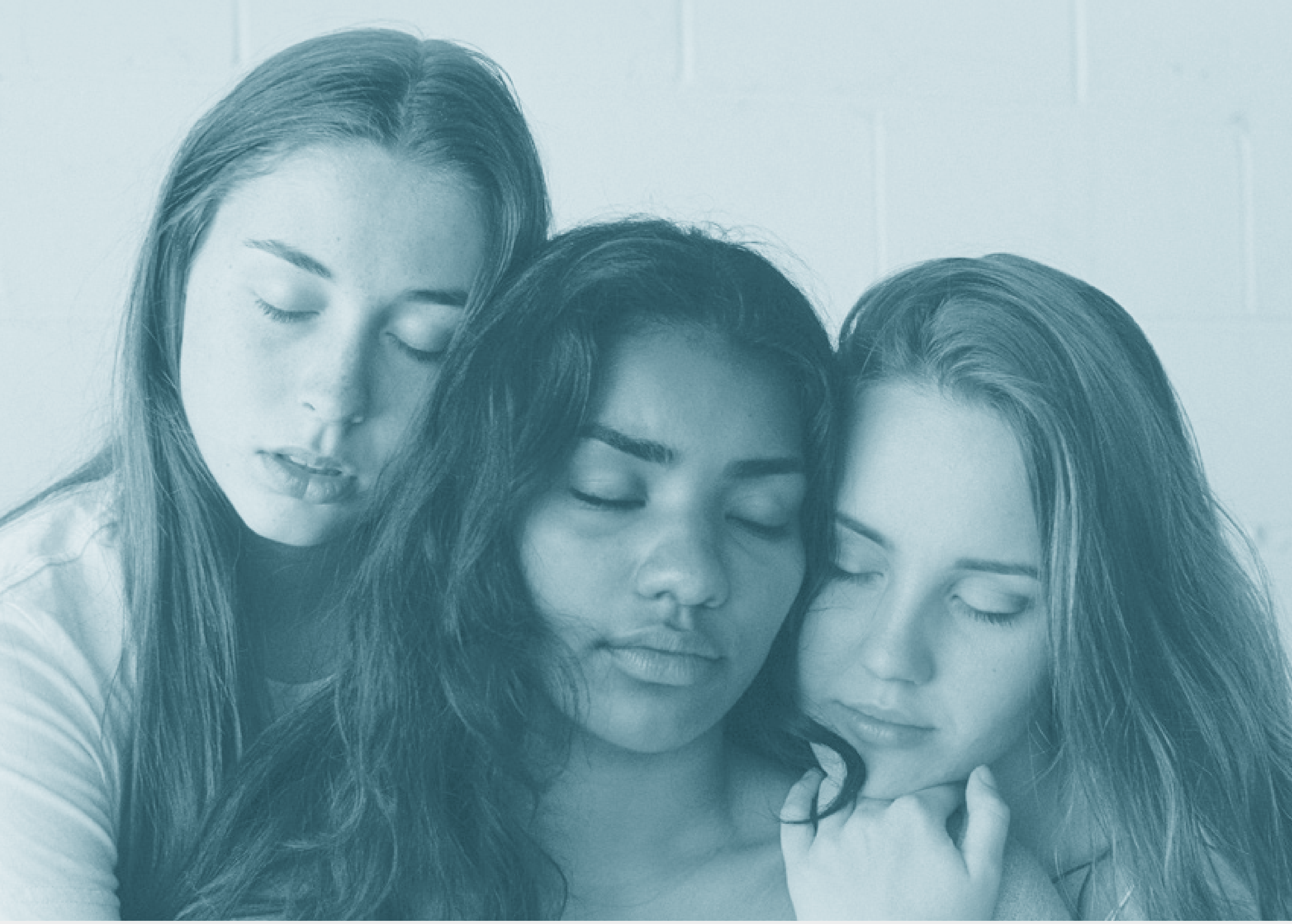

UNIVERSIDAD ICESI | OEM OBSERVATORIO PARA LA EQUIDAD DE LAS MUJERES | fundación wwb Colombia

OEM
OBSERVATORIO PARA LA EQUIDAD DE LAS MUJERES

BIOSECURITY AND ETHICS PROTOCOL

READ BEFORE STARTING THE SURVEY

Mrs. xxx, I am going to read you a short instruction that responds to a protocol of biosecurity and ethics that must be followed in this type of survey: Some of the questions below may be sensitive and it is ideal for you to be able to answer them calmly and freely. Taking this into account, and to address this situation, will have a keyword in case you feel at risk or in danger because someone in your environment listens to your responses and retaliates for giving this information. At any time you can say the NAME OF ANY FRUIT and I will assume you are in danger and will stop the questions and wait for you to give me an indication that you feel calm and safe to return to answering the survey. In case the danger is serious and imminent, I ask you to tell me the name of a fruit and hang up the call, with which I will call you again in five minutes, if you do not answer the call, proceed to notify the authorities that you are in danger.

DURING THE SURVEY

The following scenarios may be presented to you, please read and study them beforehand so that you will know what to do in case they occur.

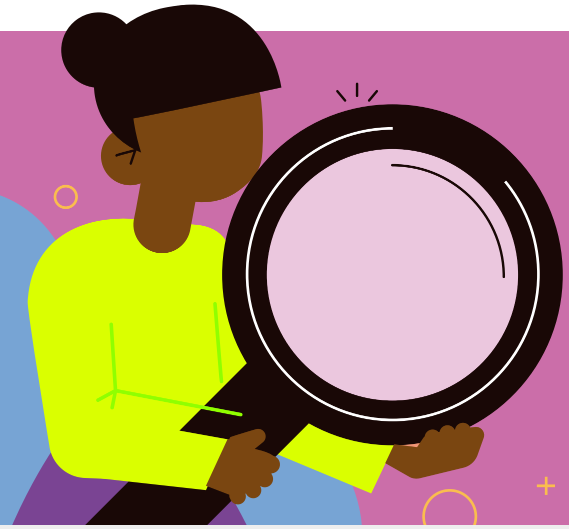

01

SCENARIO 1

THE RESPONDENT DECLARES

TO BE AT RISK

You must **hang up the call and wait 5 minutes.** Contact her again and if she does not respond you must notify the OEM.

02

SCENARIO 2

THE RESPONDENT SAYS

NOT TO BE IN A SUITABLE ENVIRONMENT TO CARRY

OUT THE SURVEY.

You must ask the respondent to **provide you with a time** when she considers that she can answer the survey calmly, then hang up the call and contact her at the time she indicates.

03

SCENARIO 3

THE RESPONDENT CRIES

ON A QUESTION,

OR AT SOME TIME IN THE SURVEY.

You must explain to **her that the survey can be ended at any time, and that you can recontact her when she feels better.** In addition, you will provide the email address of the OEM where you can give her a route for psychological care (info@oemcolombia.com) or the cell phone number 3178985248.

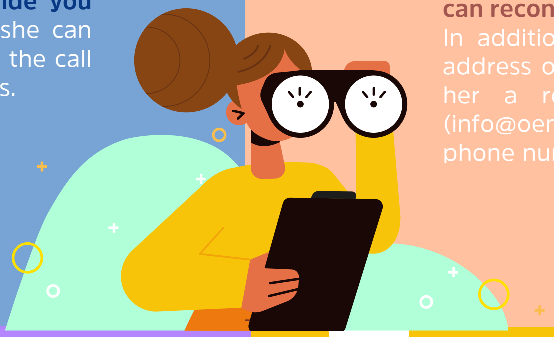

04

SCENARIO 4

THE RESPONDENT DEVIATES FROM THE QUESTION

ASK AND TELL AN ANECDOTE OR STORY.

It is important that you be empathetic with life stories, but if you consider that the anecdote is too long, you can interrupt the respondent and tell her that it is important to complete the survey and if you see that the story is traumatic, you can tell her that if she wishes, she can communicate with the OEM email (info@oemcolombia.com) or call the phone number (3178985248) to receive a route of psychological care.

05

SCENARIO 5

THE RESPONDENT

REMAINS SILENT

You must repeat the initial sentence in which you indicate: **In case you feel at risk or in danger, I ask you to please tell me the name of a fruit** and I will wait for you to give me an indication again that you are safe to continue with the survey process. In the event that the danger is serious and imminent, I ask that you please tell me the name of a fruit and hang up the call, whereupon I will proceed to call you back in five minutes, if you do not answer the call, proceed to notify the authorities that you are in danger.

AT THE END OF THE SURVEY

If you consider that the respondent may be going through a dangerous moment or needs attention, please save that number in your database and indicate this case to the OEM to contact this respondent and offer the relevant alternatives.
