# Supplementary material for: Gender-based violence: Statistical data for four Colombian municipalities
Source: Data Brief. 2022 May 28;43:108320. doi: 10.1016/j.dib.2022.108320 (PMC9189776; doi:10.1016/j.dib.2022.108320)
Supplement: Supplementary file 2 [file mmc2.pdf]

ICESI – Violence		**Centro Nacional de Consultoría S.A.**<br>Calle 82 # 6-51 Bogotá<br>Phone: 339 4888	**GENDER-BASED VIOLENCE - OBSERVATORIO PARA LA EQUIDAD DE LAS MUJERES CALI – JAMUNDÍ – YUMBO - BUENAVENTURA**

This questionnaire is the basis for the design of the digital questionnaire that was filled out by the pollsters

First contact greeting" Good morning. My name is ______ of the Centro Nacional de Consultoría, a private company dedicated to market, social and public opinion research, and work for the Observatorio para la Equidad de las Mujeres, of the ICESI university and WWB Foundation Colombia. We are conducting a citywide survey of women over the age of 18 on issues associated with family life, the economy, public participation and gender-based violence".
"Please can you connect me with a woman over the age of 18 living in the home ".
Yes_____ Not present __________ thank and close the survey.

Greet the woman of the home "Good morning. My name is ______ of the Centro Nacional de Consultoría, a private company dedicated to market, social and public opinion research, and work for the Observatorio para la Equidad de las Mujeres, of the ICESI university and WWB Foundation Colombia. We are conducting a citywide survey of women over the age of 18 on issues associated with family life, the economy, public participation and gender-based violence".

This information will be used only for statistical and academic purposes to influence policies in favor of women's equality. This is in accordance with Law 1581 of 2012 on the protection of personal data. All information that you provide us will be kept strictly confidential and will not be disclosed to others. Your participation in this interview is voluntary and if any question arises that you do not want to answer, let me know and I will continue with the following questions.

We hope to count on you, since your participation is very important for this process. ¿Do you agree? With these details, I ask for your authorization to take your data and do the survey, which will take us approximately 20 minutes. Authorize: Yes______ No______

I am going to read you a short instruction that responds to a protocol of biosecurity and ethics that must be followed in this type of survey: Some of the questions below may be sensitive and it is ideal for you to be able to answer them calmly and freely. Taking this into account, and to address this situation, will have a keyword in case you feel at risk or in danger because someone in your environment listens to your responses and retaliates for giving this information. At any time you can say the NAME OF ANY FRUIT and I will assume you are in danger and will stop the questions and wait for you to give me an indication that you feel calm and safe to return to answering the survey. In case the danger is serious and imminent, I ask you to tell me the name of a fruit and hang up the call, with which I will call you again in five minutes, if you do not answer the call, proceed to notify the authorities that you are in danger

Section 1 Socio demographical information

a. ¿Could you tell me the city/area of your current residence?

76001	Cali	01
76109	Buenaventura	02
76364	Jamundí	03
76892	Yumbo	04

a1. Could you tell me please, ¿what is your commune of residence?

Commune 1	01	Commune 12	12
Commune 2	02	Commune 13	13
Commune 3	03	Commune 14	14
Commune 4	04	Commune 15	15
Commune5	05	Commune 16	16
Commune 6	06	Commune 17	17
Commune 7	07	Commune 18	18
Commune 8	08	Commune 19	19
Commune 9	09	Commune 20	20
Commune 10	10	Commune 21	21
Commune 11	11	Commune 22	22

b. Could you tell me please, ¿what is the address of your residence?

c. Could you tell me please, ¿what is the neighborhood of your home?

d. According to the receipt of electricity and public services, what is the stratum of your residence?

One	01
Two	02
Three	03
Four	04
Five	05
Six	06

1. Date of Birth

D	D	M	M	Y	Y	Y	Y

2. Depending on your culture, customs, or traditions, you identify yourself as

Afro-Colombian, Afro-descendant (black, mulatto, root, palenquera)	1
Indigineous	2
Mestiza	3
White	4
Rom o gypsy	5
Other	77
None	88

3. Sex registered at birth

Man	1
Woman	2

3.1 What is your marital status?

Single	1
Free union	2
Casada	3
Married	4
Separated / Divorced	5
Other	77

4. ¿Do you identify yourself as? **(E: Read the options, give the clarifying definition if the respondent asks for the meaning of the term)**

Woman	01
Transgender Woman (you were registered as a male, but you identify yourself as a female)	02
Transgender Man (you were registered as a woman, but you identify yourself as a man)	03
Other ¿which one? _______________	77

5. ¿What is your sexual orientation? **(E: Read the options, RU, Give the clarifying definition if the respondent asks you what sexual orientation is: It corresponds to your erotic affective attraction for another person)**

Heterosexual (for people of the opposite sex to yours, in this case men)	01
Homosexual (for people of the same sex of yours, in this case women)	02
Bisexual (for people of the opposite sex and of the same sex of yours, in this case men and women)	03
Other ___ ¿which one? ____	04
Do not know/no answer	66

6. ¿Are you studying actually?

Yes	1
No	2

7. ¿What is the highest educational level attained by you?

Educational level	cod	Educational level	cod
None	88	Bachelor's Degree incompleted	09
Elementary School completed	02	Specialization completed	10
Elementary School incompleted	03	Specialization incompleted	11
High School completed	04	Do not know/no response	99
High School incompleted	05		
Technological or Technical studies completed	06		
Technological or Technical studies incompleted	07		
Bachelor's Degree completed	08		

8. During the last month, what did you spend most of your time on?

Working	01	Go to Q11
Looking for a job	02	Continue
Studying	03	Continue
Unpaid housework (include taking care of relatives or close ones)	04	Continue
Permanently disabled for work	05	Go a Q11
Other activity	77	Continue

10. The main reason you did not work last week was …

Retired	01
Your studies take up too much of your time	02
Unpaid care work at home does not allow time for work (include taking care of relatives or close ones)	03
Cannot get a job	04
Health problems	05
You do not need to work because you receive money from rents, rentals, or the profitability of some property or investment.	06
You do not like to work or think it is not worth it.	07
Other	77

Section 2: Household

11. ¿Do you live with someone else?

Yes	01	Continue
No	02	Go to Q16

11.a. List the number of people that living in your household who share food, not including yourself.

12. Name of each person who lives in the household with you, who shares the meal (E. Do not include the respondent)

M1	
M2	
M3	
M4	
M5	
M6	
M7	
M8	
M9	
M10	

13. ¿How are they related to you? **(ASK FOR EACH PERSON WHO ANSWERED IN Q12)**

	M1	M2	M3	M4	M5	M6	M7	M8	M9	M10
Spouse	1	1	1	1	1	1	1	1	1	1
Child	2	2	2	2	2	2	2	2	2	2
Grandchild/ granddaughter	3	3	3	3	3	3	3	3	3	3
Father/Mother	4	4	4	4	4	4	4	4	4	4
Father/Mother In-law	5	5	5	5	5	5	5	5	5	5
Sibling	6	6	6	6	6	6	6	6	6	6
Other relative	7	7	7	7	7	7	7	7	7	7
Not related	8	8	8	8	8	8	8	8	8	8
Other	77	77	77	77	77	77	77	77	77	77
Do not know/no answer	66	66	66	66	66	66	66	66	66	66

14. Age. (Indicate that they are years old on the date of the call, if they are under one year of age, mark “0” (ask for each person who answered on Q12)

14.1 Sex. (**Remember sex**: man 1, woman 2, other 3) (ask for each person who answered in Q12)

	Q14	Q14.1
M1		
M2		
M3		
M4		
M5		
M6		
M7		
M8		
M9		
M10		

16. ¿Do you have or have you ever been in a relationship?

Yes	01
No	02

VIOLENCE AGAINST WOMEN (ASK EVERYONE)

(E: The interviewer should be patiently and calmly available for the following questions, avoid any type of facial, body, or verbal expression that shows any type of bias or judgment about the responses of the respondent. In case of silence, allow reasonable time to respond. If she does not wish to answer a question, remind the respondent that the responses are confidential and anonymous, without pressuring her to do so. Make sure that these questions are answered without the presence of other people. (Do not read in any question the option of does not want to answer).

The questions that follow are associated with interpersonal relationships throughout your life, especially with your partner. I know that some of these questions are very personal, but I assure you that your answers are completely confidential and will be of great importance to understand some aspects of violence against women.

Sección 3.1 Psychological violence

17. Someone has made comments to belittle you or diminish your self-esteem (e.g., You are a brute, you are good for nothing, you don't do anything right, etc.)?

Yes	1	Continue
No	2	Go yo Q18
Do not know/no answer	66	Go to Q18

17.1. ¿Who? **(The respondent can answer up to 3)**

Any of your parents	01
Any of your children	02
Other relative	03
Your current partner (**Exclude only those who answered *no* in Q16)**	04
Your former partner	05
A non-relative acquaintance	06
A stranger	07
Do not know/no answer	66

17.2. ¿Has it happened several times or just once?

Many times	1
One time	2
Do not know/no answer	66

17.3. ¿When was the last time this happened to you?

More than 10 years ago	01
More than a year ago	02
During the last year	03
During the last month	04
During the last week	05
Do not know/no answer	66

18. ¿Has anyone made unwanted comments to you about your appearance or body that have made you feel uncomfortable or insecure?

Yes	1 continue
No	2 Go to Q21
Do not know/no answer	66 Go to Q21

18.1 ¿Who? **(The respondent can answer up to 3)**

Any of your parents	01
Any of your children	02
Other relative	03
Your current partner (**Exclude only those who answered *no* in Q16)**	04
Your former partner	05
A non-relative acquaintance	06
A stranger	07
Do not know/no answer	66

18.2. ¿Has it happened several times or just once?

Many times	1
One time	2
Do not know/no answer	66

18.3. ¿When was the last time this happened to you?

More than 10 years ago	01
More than a year ago	02
During the last year	03
During the last month	04
During the last week	05
Do not know/no answer	66

21. ¿Has anyone sent you messages, posted comments with non-consensual sexual innuendos via cell phone, email, or social networks, published photographs or private information about you without your consent, on networks, or threatened you with doing so, etc.?

Yes	1	Continue
No	2	Go to Q22
Do not know/no answer	66	Go to Q22

21.1. ¿Who? **(The respondent can answer up to 3)**

Any of your parents	01
Any of your children	02
Other relative	03
Your current partner (**Exclude only those who answered *no* in Q16)**	04
Your former partner	05
A non-relative acquaintance	06
A stranger	07
Do not know/no answer	66

21.2¿Has it happened several times or just once?

Many times	1
One time	2
Do not know/no answer	66

21.3 ¿When was the last time this happened to you?

More than 10 years ago	01
More than a year ago	02
During the last year	03
During the last month	04
During the last week	05
Do not know/no answer	66

22 ¿Has anyone stalked or followed you when you left school, home or work?

Yes	1	Continue
No	2	Go to Q23
Do not know/no answer	66	Go to Q23

22.1. ¿Who? **(The respondent can answer up to 3)**

Any of your parents	01
Any of your children	02
Other relative	03
Your current partner (**Exclude only those who answered *no* in Q16)**	04
Your former partner	05
A non-relative acquaintance	06
A stranger	07
Do not know/no answer	66

22.2 ¿Has it happened several times or just once?

Many times	1
One time	2
Do not know/no answer	66

22.3¿When was the last time this happened to you?

More than 10 years ago	01
More than a year ago	02
During the last year	03
During the last month	04
During the last week	05
Do not know/no answer	66

23 ¿Has anyone prevented you from meeting with friends or tried to limit contact with your family?

Yes	1	Continue
No	2	Go to Q24
Do not know/no answer	66	Go to Q24

23.1¿Who? **(The respondent can answer up to 3)**

Any of your parents	01
Any of your children	02
Other relative	03
Your current partner (**Exclude only those who answered *no* in Q16)**	04
Your former partner	05
A non-relative acquaintance	06
A stranger	07
Do not know/no answer	66

23.2¿Has it happened several times or just once?

Many times	1
One time	2
Do not know/no answer	66

23.3¿When was the last time this happened to you?

More than 10 years ago	01
More than a year ago	02
During the last year	03
During the last month	04
During the last week	05
Do not know/no answer	66

24. ¿Has anyone threatened you and/or taken you away from your loved ones or pets (include if your loved ones or pets have been hurt)?

Yes	1	Continue
No	2	Go to Q25
Do not know/no answer	66	Go toQ25

24.1. ¿Who? **(The respondent can answer up to 3)**

Any of your parents	01
Any of your children	02
Other relative	03
Your current partner (**Exclude only those who answered *no* in Q16)**	04
Your former partner	05
A non-relative acquaintance	06
A stranger	07
Do not know/no answer	66

24.2¿Has it happened several times or just once?

Many times	1
One time	2
Do not know/no answer	66

24.3¿When was the last time this happened to you?

More than 10 years ago	01
More than a year ago	02
During the last year	03
During the last month	04
During the last week	05
Do not know/no answer	66

Section 3.2 Economic violence

25 ¿Has anyone restricted your access to resources such as money or food?

Yes	1	Continue
No	2	Go to Q26
Do not know/no answer	66	Go to Q26

25.1. ¿Who? **(The respondent can answer up to 3)**

Any of your parents	01
Any of your children	02
Other relative	03
Your current partner (**Exclude only those who answered *no* in Q16)**	04
Your former partner	05
A non-relative acquaintance	06

A stranger	07
Do not know/no answer	66

25.2¿Has it happened several times or just once?

Many times	1
One time	2
Do not know/no answer	66

25.3 ¿When was the last time this happened to you?

More than 10 years ago	01
More than a year ago	02
During the last year	03
During the last month	04
During the last week	05
Do not know/no answer	66

26 ¿Has anyone appropriated or taken money or goods (property, land) from you?

Yes	1	Continue
No	2	Go to Q27
Do not know/no answer	66	Go tu Q27

26.1. ¿Who? **(The respondent can answer up to 3)**

Any of your parents	01
Any of your children	02
Other relative	03
Your current partner (**Exclude only those who answered *no* in Q16)**	04
Your former partner	05
A non-relative acquaintance	06
A stranger	07
Do not know/no answer	66

26.2¿Has it happened several times or just once?

Many times	1
One time	2
Do not know/no answer	66

26.3 ¿When was the last time this happened to you?

More than 10 years ago	01
More than a year ago	02
During the last year	03
During the last month	04
During the last week	05
Do not know/no answer	66

27 ¿Has anyone forbidden you to study or work or start a business?

Yes	1	Continue
No	2	Go to Q28
Do not know/no answer	66	Go to Q28

27.1. ¿Who? **(The respondent can answer up to 3)**

Any of your parents	01
Any of your children	02
Other relative	03
Your current partner (**Exclude only those who answered *no* in Q16)**	04
Your former partner	05
A non-relative acquaintance	06
A stranger	07
Do not know/no answer	66

27.2 ¿Has it happened several times or just once?

Many times	1
One time	2
Do not know/no answer	66

27.3 ¿When was the last time this happened to you?

More than 10 years ago	01
More than a year ago	02
During the last year	03
During the last month	04
During the last week	05
Do not know/no answer	66

28 ¿Has anyone barred you from claiming any allowances or if you have received them have they been taken away?

Yes	1	Continue
No	2	Go to Q29
Do not know/no answer	66	Go to Q29

28.1. ¿Who? **(The respondent can answer up to 3)**

Any of your parents	01
Any of your children	02
Other relative	03
Your current partner (**Exclude only those who answered *no* in Q16)**	04
Your former partner	05
A non-relative acquaintance	06
A stranger	07
Do not know/no answer	66

28.2¿Has it happened several times or just once?

Many times	1
One time	2
Do not know/no answer	66

28.3 ¿When was the last time this happened to you?

More than 10 years ago	01
More than a year ago	02
During the last year	03
During the last month	04
During the last week	05
Do not know/no answer	66

29 ¿Has anyone hidden or controlled your documents with which you could have access to resources such as ID cards, bank cards, pass, among others, or has prohibited you from having a bank account?

Yes	1	Continue
No	2	Go to Q31
Do not know/no answer	66	Go tu Q27

29.1.¿Quién o quiénes? **(The respondent can answer up to 3)**

Any of your parents	01
Any of your children	02
Other relative	03
Your current partner (**Exclude only those who answered *no* in Q16)**	04
Your former partner	05
A non-relative acquaintance	06
A stranger	07
Do not know/no answer	66

29.2 ¿Has it happened several times or just once?

Many times	1
One time	2
Do not know/no answer	66

29.3¿When was the last time this happened to you?

More than 10 years ago	01
More than a year ago	02
During the last year	03
During the last month	04
During the last week	05
Do not know/no answer	66

31. ¿Has anyone threatened to throw you out of the house if you do not agree to their requests?

Yes	1	Continue
No	2	Go to Q32
During the last week	66	Go to Q32

31.1. ¿Who **(The respondent can answer up to 3)**

Any of your parents	01
Any of your children	02
Other relative	03
Your current partner (**Exclude only those who answered *no* in Q16)**	04
Your former partner	05
A non-relative acquaintance	06
A stranger	07
Do not know/no answer	66

31.2¿Has it happened several times or just once?

Many times	1
One time	2
Do not know/no answer	66

31.3 ¿When was the last time this happened to you?

More than 10 years ago	01
More than a year ago	02
During the last year	03
During the last month	04
During the last week	05
Do not know/no answer	66

32. ¿Has anyone demanded that you hand over the money you earned?

Yes	1	Continue
No	2	Go to Q33
Do not know/no answer	66	Go to Q33

32.1. ¿Who? **(The respondent can answer up to 3)**

Any of your parents	01
Any of your children	02
Other relative	03
Your current partner (**Exclude only those who answered *no* in Q16)**	04
Your former partner	05
A non-relative acquaintance	06
A stranger	07
Do not know/no answer	66

32.2 ¿Has it happened several times or just once?

Many times	1
One time	2
Do not know/no answer	66

32.3 ¿When was the last time this happened to you?

More than 10 years ago	01
More than a year ago	02
During the last year	03
During the last month	04
During the last week	05
Do not know/no answer	66

33. Did your partner or ex-partner put all assets in his name (e.g., house, car, cell phones), or has he sold your assets or household assets without your consent? **(only ask if the respondent answered 1(yes) in Q16)**

Yes	1	Continue
No	2	Go to Q34a
No apply	3	Go to Q34a
Do not know/no answer	66	Go to Q34a

33.1. ¿Has it happened several times or just once?

Many times	1
One time	2
Do not know/no answer	66

33.2 ¿When was the last time this happened to you?

More than 10 years ago	01
More than a year ago	02
During the last year	03
During the last month	04
During the last week	05
Do not know/no answer	66

34a. ¿Do you have children who are financially dependent on you?

Yes	1 Go to Q34
No	2 Go to Q40

34. ¿Has your partner or ex-partner - having the money - refused to pay your children's school expenses? **(Taking into account: Only ask if the respondent answered 1(yes) in Q34a)**

Yes	1	Continue
No	2	Go to Q35
Do not know/no answer	66	Go to Q35

34.1. ¿Has it happened several times or just once?

Many times	1
One time	2
Do not know/no answer	66

34.2 ¿When was the last time this happened to you?

More than 10 years ago	01
More than a year ago	02
During the last year	03
During the last month	04
During the last week	05
Do not know/no answer	66

35. ¿Has your current or ex-partner-having the money-refused to buy clothes or household goods for your children? **(Taking into account: Only ask if the respondent answered 1(yes) in Q34a)**

Yes	1	Continue
No	2	Go to Q36
Do not know/no answer	66	Go to Q36

35.1. ¿Has it happened several times or just once?

Many times	1
One time	2
Do not know/no answer	66

35.2¿When was the last time this happened to you?

More than 10 years ago	01
More than a year ago	02
During the last year	03
During the last month	04
During the last week	05
Do not know/no answer	66

36. ¿Has your current or former partner-having the money-refused to buy food for your children?

Yes	1	Continue
No	2	Go to Q37
Do not know/no answer	66	Pase a P37

36.1. ¿Has it happened several times or just once?

Many times	1
One time	2
Do not know/no answer	66

36.2 ¿When was the last time this happened to you?

More than 10 years ago	01
More than a year ago	02
During the last year	03
During the last month	04
During the last week	05
Do not know/no answer	66

37. ¿Has your current or ex-partner - having the money - refuse to pay child support? **(Taking into account: Only ask if the respondent answered 1(yes) in Q34a)**

Yes	1	Continue
No	2	Go to Q40
Do not know/no answer	66	Go to Q40

37.1. ¿Has it happened several times or just once?

Many times	1
One time	2
Do not know/no answer	66

37.2 ¿When was the last time this happened to you?

More than 10 years ago	01
More than a year ago	02
During the last year	03
During the last month	04
During the last week	05
Do not know/no answer	66

40. ¿Has your current or ex-partner monitor what you do at work (time of arrival and departure, activities you do, relationships with co-workers, etc.)? (**only ask if the respondent answered 1(yes) in Q16)**

Yes	1
No	2
Do not know/no answer	66

41. ¿Has your current or former partner make important decisions about how to use money in the household without consulting you or taking your opinion into account? **(only ask if the respondent answered 1(yes) in Q16)**

Yes	1
No	2
Do not know/no answer	66

42. ¿Has your current or former partner destroyed your belongings? **(only ask if the respondent answered 1(yes) in Q16)**

Yes	1
No	2
Do not know/no answer	66

Section 3.3 Sexual Violence

44. Has anyone ever shown you their private parts or touch them in front of you?

Yes	1	Continue
No	2	Go to Q45
Do not know/no answer	66	Go to Q45

44.1. ¿Who? **(The respondent can answer up to 3)**

Any of your parents	01
Any of your children	02
Other relative	03
Your current partner (**Exclude only those who answered *no* in Q16)**	04
Your former partner	05
A non-relative acquaintance	06
A stranger	07
Do not know/no answer	66

44.2¿Has it happened several times or just once?

Many times	1
One time	2
Do not know/no answer	66

44.3 ¿When was the last time this happened to you?

More than 10 years ago	01
More than a year ago	02
During the last year	03
During the last month	04
During the last week	05
Do not know/no answer	66

45 ¿Have you been forced to watch sexual or pornographic scenes or acts?

Yes	1	Continue
No	2	Go to Q46
Do not know/no answer	66	Go to Q46

45.1. ¿Who? **(The respondent can answer up to 3)**

Any of your parents	01
Any of your children	02
Other relative	03
Your current partner (**Exclude only those who answered *no* in Q16)**	04
Your former partner	05
A non-relative acquaintance	06
A stranger	07
Do not know/no answer	66

45.2 ¿Has it happened several times or just once?

Many times	1
One time	2
Do not know/no answer	66

45.3 ¿When was the last time this happened to you?

More than 10 years ago	01
More than a year ago	02
During the last year	03
During the last month	04
During the last week	05
Do not know/no answer	66

46. ¿Have you been propositioned or approached for sex in exchange for any benefits?

Yes	1	Continue
No	2	Go to Q47
Do not know/no answer	66	Go to Q47

46.1.¿Who? **(The respondent can answer up to 3)**

Any of your parents	01
Any of your children	02
Other relative	03
Your current partner (**Exclude only those who answered *no* in Q16)**	04
Your former partner	05
A non-relative acquaintance	06
A stranger	07
Do not know/no answer	66

46.2. ¿Has it happened several times or just once?

Many times	1
One time	2
Do not know/no answer	66

46.3.¿When was the last time this happened to you?

More than 10 years ago	01
More than a year ago	02
During the last year	03
During the last month	04
During the last week	05
Do not know/no answer	66

47. ¿Have you been punished, mistreated, assaulted, or taken any action against you because you refused to have sex?

Yes	1	Continue
No	2	Go to Q48
Do not know/no answer	66	Go to Q48

47.1. ¿Who? **(The respondent can answer up to 3)**

Any of your parents	01
Any of your children	02
Other relative	03
Your current partner (**Exclude only those who answered *no* in Q16)**	04
Your former partner	05
A non-relative acquaintance	06
A stranger	07
Do not know/no answer	66

47.2. ¿Has it happened several times or just once?

Many times	1
One time	2
Do not know/no answer	66

47.3. ¿When was the last time this happened to you?

More than 10 years ago	01
More than a year ago	02
During the last year	03
During the last month	04
During the last week	05
Do not know/no answer	66

48. ¿Have you been groped, touched, kissed, or approached, touched, kissed intentionally and without your consent?

Yes	1	Continue
No	2	Go to Q49
Do not know/no answer	66	Go to Q49

48.1. ¿Who? **(The respondent can answer up to 3)**

Any of your parents	01
Any of your children	02
Other relative	03
Your current partner (**Exclude only those who answered *no* in Q16)**	04
Your former partner	05
A non-relative acquaintance	06
A stranger	07
Do not know/no answer	66

48.2.¿Has it happened several times or just once?

Many times	1
One time	2
Do not know/no answer	66

48.3. ¿When was the last time this happened to you?

More than 10 years ago	01
More than a year ago	02
During the last year	03
During the last month	04
During the last week	05
Do not know/no answer	66

49. ¿Have you been forced to have sex against your will, even if you initially consented to the relationship and then changed your mind?

Yes	1	Continue
No	2	Go to Q50
Do not know/no answer	66	Go to Q50

49.1. ¿Who? **(The respondent can answer up to 3)**

Any of your parents	01
Any of your children	02
Other relative	03
Your current partner (**Exclude only those who answered *no* in Q16)**	04
Your former partner	05
A non-relative acquaintance	06
A stranger	07
Do not know/no answer	66

49.2. ¿Has it happened several times or just once?

Many times	1
One time	2
Do not know/no answer	66

49.3. ¿When was the last time this happened to you?

More than 10 years ago	01
More than a year ago	02
During the last year	03
During the last month	04
During the last week	05
Do not know/no answer	66

Sección 3.4 Physical Violence

50. ¿Have you been kicked, hit with a fist, pinched, had your hair pulled, pushed, shoved, pulled, slapped, or thrown an object?

Yes	1	Continue
No	2	Go to Q51
Do not know/no answer	66	Go to Q51

50.1. ¿Who? **(The respondent can answer up to 3)**

Any of your parents	01
Any of your children	02
Other relative	03
Your current partner (**Exclude only those who answered *no* in Q16)**	04
Your former partner	05
A non-relative acquaintance	06
A stranger	07
Do not know/no answer	66

50.2. ¿Has it happened several times or just once?

Many times	1
One time	2
Do not know/no answer	66

50.3. ¿When was the last time this happened to you?

More than 10 years ago	01
More than a year ago	02
During the last year	03
During the last month	04
During the last week	05
Do not know/no answer	66

51. ¿Have you been attacked or assaulted with a knife, firearm or chemicals?

Yes	1	Continue
No	2	Go to Q52
Do not know/no answer	66	Go to Q52

51.1. ¿Who? **(The respondent can answer up to 3)**

Any of your parents	01
Any of your children	02
Other relative	03
Your current partner (**Exclude only those who answered *no* in Q16)**	04
Your former partner	05
A non-relative acquaintance	06
A stranger	07
Do not know/no answer	66

51.2. ¿Has it happened several times or just once?

Many times	1
One time	2
Do not know/no answer	66

51.3. ¿When was the last time this happened to you?

More than 10 years ago	01
More than a year ago	02
During the last year	03
During the last month	04
During the last week	05
Do not know/no answer	66

52. ¿Do you believe that your home is a place free of violence? (free from violence such as those mentioned above)

Yes	1
No	2
Do not know/no answer	66

Sección 3.5 Workplace violence–Workplace harrasment

53. ¿Have you been paid less than a fellow man who does the same job or has the same position as you?

Yes	1
No	2
Do not know/no answer	66

54. ¿Have you had fewer opportunities for promotion compared to men?

Yes	1
No	2
Do not know/no answer	66

55. ¿Because you are pregnant, or because you have small children, you have not been hired, your salary has been lowered or you have been fired?

Yes	1
No	2
Do not know/no answer	66

56. ¿Because of your marital status, gender identity, or sexual orientation, you were not hired, your salary was lowered or you were fired?

Yes	1
No	2
Do not know/no answer	66

58. ¿Have you ever been asked to take a pregnancy test as a requirement for employment or continuation of employment?

Yes	1
No	2
Do not know/no answer	66

59. ¿Have you been denied maternity leave law or breastfeeding leave?

Yes	1
No	2
Not apply– Never been pregnant	3
Do not know/no answer	66

61. ¿Have you ever been told that because you are a woman you are not suitable for a job?

Yes	1
No	2
Do not know/no answer	66

62. ¿Have you been forced to work longer hours than your male colleagues?

Yes	1
No	2
Do not know/no answer	66

63. ¿In your job, have you had to perform unpaid care work? example: cooking, washing, caring for children, caring for older adults, ¿etc.?

Yes	1
No	2
Do not know/no answer	66

64. **¿Have you ever reported any of these situations of violence?? (Only the respondent that answered 1(yes) in Q17, Q18, Q21, Q22, Q23, Q24. Q25, Q26, Q27, Q28, Q29, Q31, Q33, Q34, Q35, Q36, Q37, Q40, Q41, Q42, Q44, Q45, Q46, Q47, Q48, Q49, Q50, Q51, Q53, Q54, Q55, Q56, Q58, Q59**

Yes	1
No	2

Section 6: Financial Autonomy

In this section we are going to talk about the financial issues, please remember that your answers are confidential

65.1 In the last month, adding salary, working overtime and benefits, ¿how much was your income from work? (only as kif the respondent answered yes (1) in Q8)

Less than 1 minimum wage (981 thousand approx.)	02
Between 1 and 2 minimum wages (approx. 981 thousand and 1,963 thousandCOP).	03
Between 2 and 3 minimum wages (1,963 thousand and 2,944 thousand COP approx.)	04
Between 3 and 4 minimum wages (2.944 thounsand and 3.926 thousand COP approx.)	05
Between 4 and 5 minimum wages (Between 3,926 thousand and 4,908 thousand COP approx.)	06
More than 5 minimum wages (More than 4,908 thousand COP approx.))	07
Does not apply	99

In this section we are going to talk about the financial issues, please remember that your answers are confidential (only ask if the respondent answered yes (1) in Q8)

65.2 ¿What are you doing to obtain resources for your retirement? (**E: read the following options)**

Makes payments to a mandatory pension fund	01
Makes payments to a voluntary pension fund	02
Save	03
Make investments	04
Pays for insurance	05
Prepares her children to support her in old age	06
Already retired	07
Nothing	08
Other	09

65.3 In the last month, ¿how much money did you receive in retirement pensions? (only ask if the respondent answerd 07 in Q65.2)

Less than 1 minimum wage (981 thousand COP approx.)	01
Between 1 and 2 minimum wages (approx. 981 thousand and 1,963 thousand COP approx.)	02
Between 2 and 3 minimum wages (1,963 thousand and 2,944 thousand COP approx.)	03
Between 3 and 4 minimum wages (2.944 thounsand and 3.926 thousand COP approx.)	04
Between 4 and 5 minimum wages (Between 3,926 thousand and 4,908 thousand COP approx.)	05
More than 5 minimum wages (More than 4,908 thousand COP approx.))	06
Do not know/no answer	99

65.4 ¿Which of the following government subsidies did you receive during the last month?

Ingreso solidario	01
Colombia Mayor	02
Familias en acción	03
Jóvenes en acción	04
Other	77
None	88

65.5. In the last month, ¿how much money did you receive from child support, family or partner support or foreign remittances?

None	01
Less than 1 minimum wage (981 thousand COP approx.)	02
Between 1 and 2 minimum wages (approx. 981 thousand and 1,963 thousand COP approx.)	03
Between 2 and 3 minimum wages (1,963 thousand and 2,944 thousand COP approx.)	04
Between 3 and 4 minimum wages (2.944 thounsand and 3.926 thousand COP approx.)	05
Between 4 and 5 minimum wages (Between 3,926 thousand and 4,908 thousand COP approx.)	06
More than 5 minimum wages (More than 4,908 thousand COP approx.))	07
Do not know/no answer	99

65. In the last month, ¿how much money did you receive from rents, profits or dividends from investments or lotteries?

None	01
Menos de 1 SMLV (981.657 aprox.)	02
Entre 1 y 2 SMLV (981.658 y 1.963.314 aprox.)	03
Entre 2 y 3 SMLV (1.963.315 Y 2.944.971aprox.)	04
Entre 3 y 4 SMLV (Entre 2.944.972 y 3.926.628 aprox.)	05
Entre 4 y 5 SMLV (Entre 3.926.629 y 4.908.285 aprox.)	06
Más de 5 SMLV (Más de 4.908.286)	07
No sabe, no responde	99

**66. Property type**		Yes	No
66.1	¿Do you have any residential real estate (house, apartment, etc.) or plot of land-titled in your name or with someone else?	01	02
66.2	¿Do you have any vehicle (motorcycle or car) for personal use title in your name or with someone else?	01	02
66.3	¿Do you have any personal property (cab, automobiles or machinery) for commercial purposes titled in your name or with anyone else?	01	02
66.6	¿Do you have biological assets (cows, pigs, chickens, etc.) owned or co-owned?	01	02

67. ¿Who primarily decides how your monthly or daily income is spent?

You	01
Your current partner	02
You and someone else	03
Someone other than your partner	04
not apply	99

68. ¿How much of your income do you spend on household expenditure??

Nothing	01
Less than half	02
Half	03
More than half	04
All	05

69. ¿Who is the principal responsible for covering your personal expenses? (beauty items, personal grooming) **(only ask if the respondent answered is from 01 Cali o 02 Buenaventura)**

No one	01
A member of your family or your partner	02
You and someone else	03
You	04

70. Who is principally responsible for household expenses? (rent payment, services etc.)?

Covered by state subsidies	01
A member of your family or your partner	02
You and someone else	03
You	04

71. Who decides on household spending and investments in real estate or vehicles? **(only ask if the respondent answered is from 01 Cali o 02 Buenaventura)**

A member of your family or your partner	01
You and someone else	02
You	03

72. If you had an emergency today that required an expenditure close to 1 minimum wage (981 thousand pesos), how would you get most of the money?

Informal credit "drop to drop"	01
Borrowing money from a bank, using a credit card, etc.	02
Borrowing money from family or friends	03
A gift of money from a partner or relative	04
An advance of your salary	05
Sale or pawning of any of your belongings	06
Use your savings	07
Other	77

73. ¿Do you save money?

Yes	1	Go to Q74_1
No	2	Go to Q75

74.1 Indicate all the ways you save

Keep your money at home	01
Family and community savings groups (chains)	02
Savings in banks, cooperatives or other entities	03
Other	77

74.2.¿How much of your income do you save?

Save what's leftover after paying your expenses	01
Saves less than a quarter of your income	02
Half	03
More than half	04

75. ¿Do you have paying debts?

Yes	1	Go to Q75_1
No	2	Go to Q78

75.1. ¿Is your monetary debt informal? "drop to drop"?

Yes	1
No	2

75.2. ¿Does your debt or part of your debts correspond to a loan, credit for merchandise or catalog, sale or pawn of any of your belongings?

Yes	1
No	2

75.3. ¿Does your debt or part of your debt correspond to a bank loan, credit card?

Yes	1
No	2

75.5 ¿Does your debt or part of your debt correspond to loans from partners, relatives or friends??

Yes	1
No	2

75.7 ¿Does your debt or part of your debts correspond to other?

Yes	1 Continue
No	2 Go to Q77

75.7.1 ¿Which? _____

77. ¿What approximate amount of your income goes to paying debts??

Less than a quarter	01
Less than half	02
Half	03
More than half	04
Do not know/no answer	99

Final section

80. ¿Would you give us your email address so that in the future we can send you information about the Observatorio Para la Equidad de las Mujeres?

87. Could you give me your complete name:

88. This information will be analyzed without taking your personal data into account, unless you authorize us to include it. Would you allow us to add your name and other personal information to these responses?

Yes	1
No	2

I appreciate your collaboration on behalf of the Centro Nacional de Consultoría, the Observatorio para la Equidad de las Mujeres from ICESI's university and the Fundación WWB Colombia, remember that you can request information from the OEM of this process and your data by contacting the line 3178985248
Have a Good day
